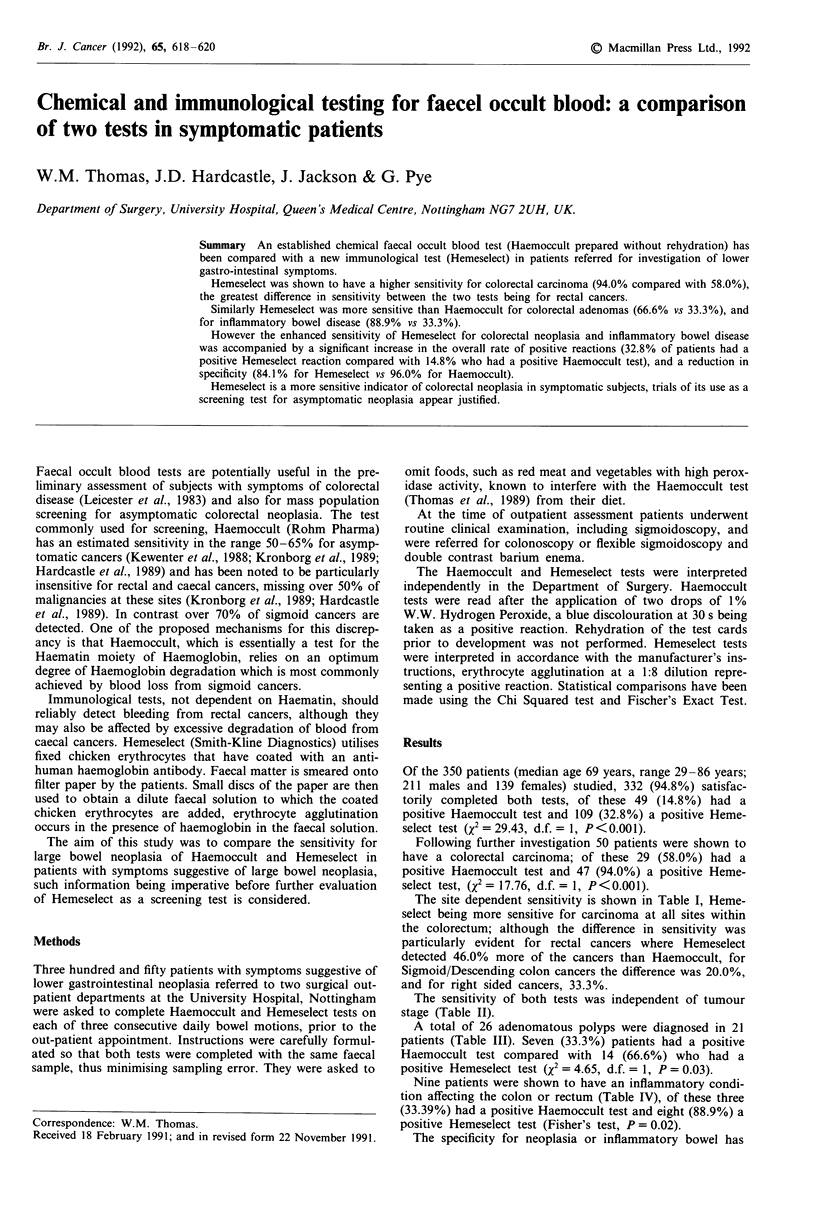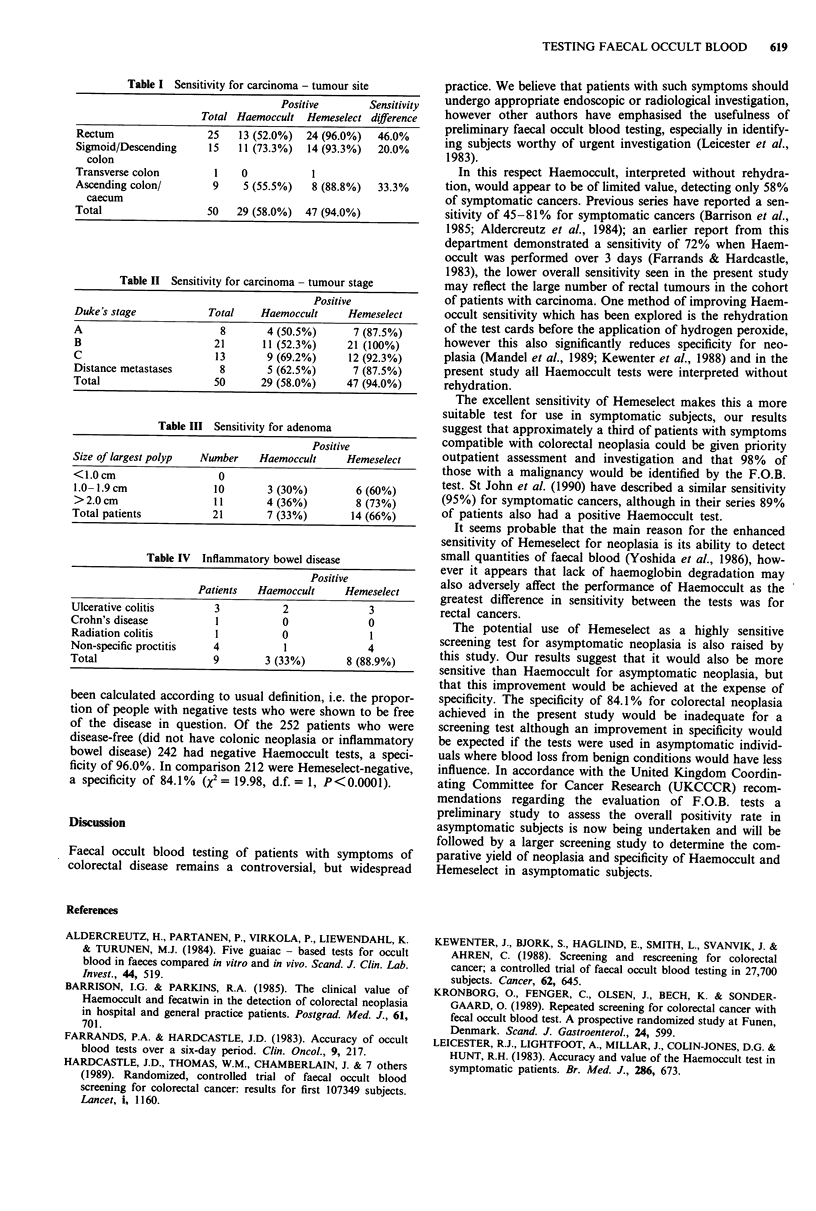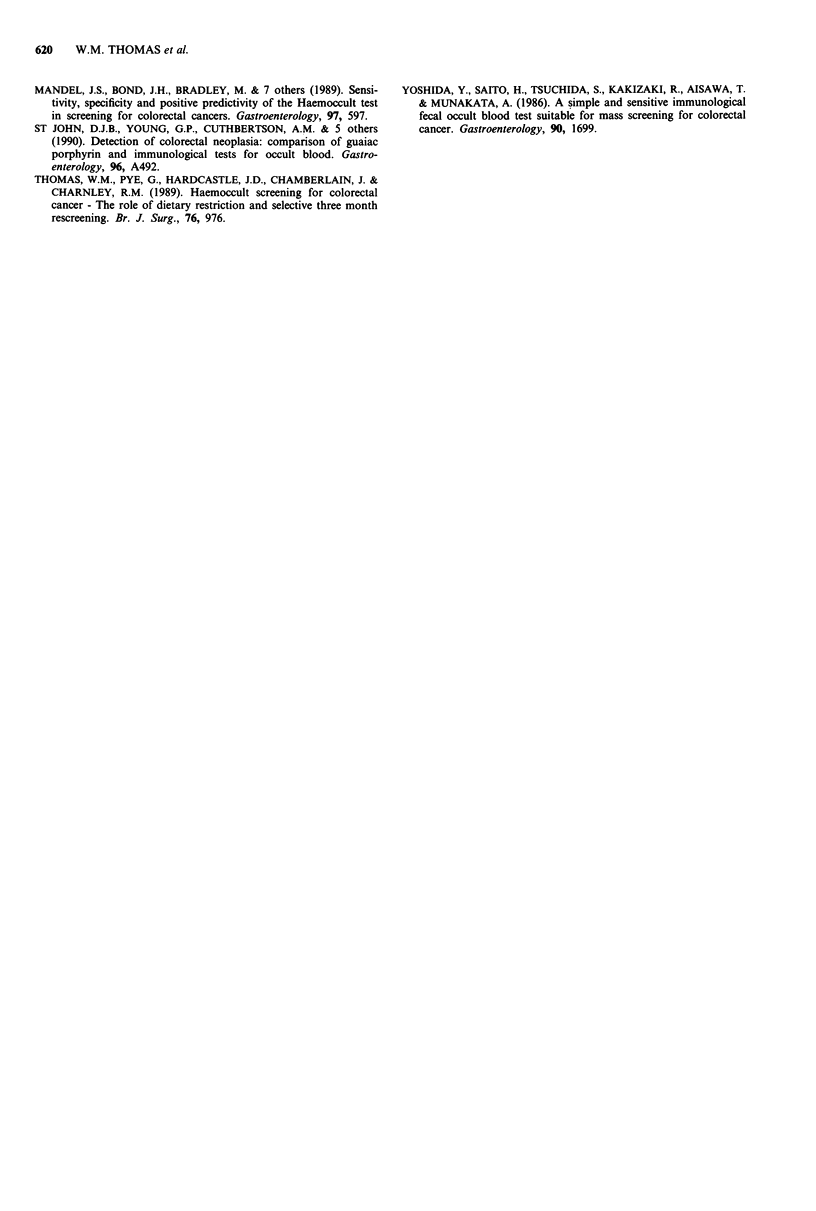# Chemical and immunological testing for faecel occult blood: a comparison of two tests in symptomatic patients.

**DOI:** 10.1038/bjc.1992.125

**Published:** 1992-04

**Authors:** W. M. Thomas, J. D. Hardcastle, J. Jackson, G. Pye

**Affiliations:** Department of Surgery, University Hospital, Nottingham, UK.

## Abstract

An established chemical faecal occult blood test (Haemoccult prepared without rehydration) has been compared with a new immunological test (Hemeselect) in patients referred for investigation of lower gastro-intestinal symptoms. Hemeselect was shown to have a higher sensitivity for colorectal carcinoma (94.0% compared with 58.0%), the greatest difference in sensitivity between the two tests being for rectal cancers. Similarly Hemeselect was more sensitive than Haemoccult for colorectal adenomas (66.6% vs 33.3%), and for inflammatory bowel disease (88.9% vs 33.3%). However the enhanced sensitivity of Hemeselect for colorectal neoplasia and inflammatory bowel disease was accompanied by a significant increase in the overall rate of positive reactions (32.8% of patients had a positive Hemeselect reaction compared with 14.8% who had a positive Haemoccult test), and a reduction in specificity (84.1% for Hemeselect vs 96.0% for Haemoccult). Hemeselect is a more sensitive indicator of colorectal neoplasia in symptomatic subjects, trials of its use as a screening test for asymptomatic neoplasia appear justified.


*Br. J. Cancer* (1992), **65**, 618–620 © Macmillan Press Ltd., 1992

### Chemical and immunological testing for faecel occult blood: a comparison of two tests in symptomatic patients

W.M. Thomas, J.D. Hardcastle, J. Jackson & G. Pye

*Department of Surgery, University Hospital, Queen's Medical Centre, Nottingham NG7 2UH, UK.*

**Summary** An established chemical faecal occult blood test (Haemoccult prepared without rehydration) has been compared with a new immunological test (Hemeselect) in patients referred for investigation of lower gastro-intestinal symptoms.

Hemeselect was shown to have a higher sensitivity for colorectal carcinoma (94.0% compared with 58.0%), the greatest difference in sensitivity between the two tests being for rectal cancers.

Similarly Hemeselect was more sensitive than Haemoccult for colorectal adenomas (66.6% *vs* 33.3%), and for inflammatory bowel disease (88.9% *vs* 33.3%).

However the enhanced sensitivity of Hemeselect for colorectal neoplasia and inflammatory bowel disease was accompanied by a significant increase in the overall rate of positive reactions (32.8% of patients had a positive Hemeselect reaction compared with 14.8% who had a positive Haemoccult test), and a reduction in specificity (84.1% for Hemeselect *vs* 96.0% for Haemoccult).

Hemeselect is a more sensitive indicator of colorectal neoplasia in symptomatic subjects, trials of its use as a screening test for asymptomatic neoplasia appear justified.

Faecal occult blood tests are potentially useful in the preliminary assessment of subjects with symptoms of colorectal disease (Leicester *et al.*, 1983) and also for mass population screening for asymptomatic colorectal neoplasia. The test commonly used for screening, Haemoccult (Rohm Pharma) has an estimated sensitivity in the range 50–65% for asymptomatic cancers (Kewenter *et al.*, 1988; Kronborg *et al.*, 1989; Hardcastle *et al.*, 1989) and has been noted to be particularly insensitive for rectal and caecal cancers, missing over 50% of malignancies at these sites (Kronborg *et al.*, 1989; Hardcastle *et al.*, 1989). In contrast over 70% of sigmoid cancers are detected. One of the proposed mechanisms for this discrepancy is that Haemoccult, which is essentially a test for the Haematin moiety of Haemoglobin, relies on an optimum degree of Haemoglobin degradation which is most commonly achieved by blood loss from sigmoid cancers.

Immunological tests, not dependent on Haematin, should reliably detect bleeding from rectal cancers, although they may also be affected by excessive degradation of blood from caecal cancers. Hemeselect (Smith-Kline Diagnostics) utilises fixed chicken erythrocytes that have coated with an anti-human haemoglobin antibody. Faecal matter is smeared onto filter paper by the patients. Small discs of the paper are then used to obtain a dilute faecal solution to which the coated chicken erythrocytes are added, erythrocyte agglutination occurs in the presence of haemoglobin in the faecal solution.

The aim of this study was to compare the sensitivity for large bowel neoplasia of Haemoccult and Hemeselect in patients with symptoms suggestive of large bowel neoplasia, such information being imperative before further evaluation of Hemeselect as a screening test is considered.

### Methods

Three hundred and fifty patients with symptoms suggestive of lower gastrointestinal neoplasia referred to two surgical out-patient departments at the University Hospital, Nottingham were asked to complete Haemoccult and Hemeselect tests on each of three consecutive daily bowel motions, prior to the out-patient appointment. Instructions were carefully formulated so that both tests were completed with the same faecal sample, thus minimising sampling error. They were asked to omit foods, such as red meat and vegetables with high peroxidase activity, known to interfere with the Haemoccult test (Thomas *et al.*, 1989) from their diet.

At the time of outpatient assessment patients underwent routine clinical examination, including sigmoidoscopy, and were referred for colonoscopy or flexible sigmoidoscopy and double contrast barium enema.

The Haemoccult and Hemeselect tests were interpreted independently in the Department of Surgery. Haemoccult tests were read after the application of two drops of 1% W.W. Hydrogen Peroxide, a blue discolouration at 30 s being taken as a positive reaction. Rehydration of the test cards prior to development was not performed. Hemeselect tests were interpreted in accordance with the manufacturer's instructions, erythrocyte agglutination at a 1:8 dilution representing a positive reaction. Statistical comparisons have been made using the Chi Squared test and Fischer's Exact Test.

### Results

Of the 350 patients (median age 69 years, range 29–86 years; 211 males and 139 females) studied, 332 (94.8%) satisfactorily completed both tests, of these 49 (14.8%) had a positive Haemoccult test and 109 (32.8%) a positive Hemeselect test ($\chi^2 = 29.43$, d.f. = 1, $P < 0.001$).

Following further investigation 50 patients were shown to have a colorectal carcinoma; of these 29 (58.0%) had a positive Haemoccult test and 47 (94.0%) a positive Hemeselect test, ($\chi^2 = 17.76$, d.f. = 1, $P < 0.001$).

The site dependent sensitivity is shown in Table I, Hemeselect being more sensitive for carcinoma at all sites within the colorectum; although the difference in sensitivity was particularly evident for rectal cancers where Hemeselect detected 46.0% more of the cancers than Haemoccult, for Sigmoid/Descending colon cancers the difference was 20.0%, and for right sided cancers, 33.3%.

The sensitivity of both tests was independent of tumour stage (Table II).

A total of 26 adenomatous polyps were diagnosed in 21 patients (Table III). Seven (33.3%) patients had a positive Haemoccult test compared with 14 (66.6%) who had a positive Hemeselect test ($\chi^2 = 4.65$, d.f. = 1, $P = 0.03$).

Nine patients were shown to have an inflammatory condition affecting the colon or rectum (Table IV), of these three (33.39%) had a positive Haemoccult test and eight (88.9%) a positive Hemeselect test (Fisher's test, $P = 0.02$).

The specificity for neoplasia or inflammatory bowel has

Correspondence: W.M. Thomas.
Received 18 February 1991; and in revised form 22 November 1991.

**Table I** Sensitivity for carcinoma – tumour site

| | *Total* | *Positive* *Haemoccult* | *Hemeselect* | *Sensitivity difference* |
|---|---|---|---|---|
| Rectum | 25 | 13 (52.0%) | 24 (96.0%) | 46.0% |
| Sigmoid/Descending colon | 15 | 11 (73.3%) | 14 (93.3%) | 20.0% |
| Transverse colon | 1 | 0 | 1 | |
| Ascending colon/ caecum | 9 | 5 (55.5%) | 8 (88.8%) | 33.3% |
| Total | 50 | 29 (58.0%) | 47 (94.0%) | |

**Table II** Sensitivity for carcinoma – tumour stage

| *Duke's stage* | *Total* | *Positive* *Haemoccult* | *Hemeselect* |
|---|---|---|---|
| A | 8 | 4 (50.5%) | 7 (87.5%) |
| B | 21 | 11 (52.3%) | 21 (100%) |
| C | 13 | 9 (69.2%) | 12 (92.3%) |
| Distance metastases | 8 | 5 (62.5%) | 7 (87.5%) |
| Total | 50 | 29 (58.0%) | 47 (94.0%) |

**Table III** Sensitivity for adenoma

| *Size of largest polyp* | *Number* | *Positive* *Haemoccult* | *Hemeselect* |
|---|---|---|---|
| <1.0 cm | 0 | | |
| 1.0–1.9 cm | 10 | 3 (30%) | 6 (60%) |
| >2.0 cm | 11 | 4 (36%) | 8 (73%) |
| Total patients | 21 | 7 (33%) | 14 (66%) |

**Table IV** Inflammatory bowel disease

| | *Patients* | *Positive* *Haemoccult* | *Hemeselect* |
|---|---|---|---|
| Ulcerative colitis | 3 | 2 | 3 |
| Crohn's disease | 1 | 0 | 0 |
| Radiation colitis | 1 | 0 | 1 |
| Non-specific proctitis | 4 | 1 | 4 |
| Total | 9 | 3 (33%) | 8 (88.9%) |

been calculated according to usual definition, i.e. the proportion of people with negative tests who were shown to be free of the disease in question. Of the 252 patients who were disease-free (did not have colonic neoplasia or inflammatory bowel disease) 242 had negative Haemoccult tests, a specificity of 96.0%. In comparison 212 were Hemeselect-negative, a specificity of 84.1% ($\chi^2 = 19.98$, d.f. = 1, $P<0.0001$).

### Discussion

Faecal occult blood testing of patients with symptoms of colorectal disease remains a controversial, but widespread practice. We believe that patients with such symptoms should undergo appropriate endoscopic or radiological investigation, however other authors have emphasised the usefulness of preliminary faecal occult blood testing, especially in identifying subjects worthy of urgent investigation (Leicester *et al.*, 1983).

In this respect Haemoccult, interpreted without rehydration, would appear to be of limited value, detecting only 58% of symptomatic cancers. Previous series have reported a sensitivity of 45–81% for symptomatic cancers (Barrison *et al.*, 1985; Aldercreutz *et al.*, 1984); an earlier report from this department demonstrated a sensitivity of 72% when Haemoccult was performed over 3 days (Farrands & Hardcastle, 1983), the lower overall sensitivity seen in the present study may reflect the large number of rectal tumours in the cohort of patients with carcinoma. One method of improving Haemoccult sensitivity which has been explored is the rehydration of the test cards before the application of hydrogen peroxide, however this also significantly reduces specificity for neoplasia (Mandel *et al.*, 1989; Kewenter *et al.*, 1988) and in the present study all Haemoccult tests were interpreted without rehydration.

The excellent sensitivity of Hemeselect makes this a more suitable test for use in symptomatic subjects, our results suggest that approximately a third of patients with symptoms compatible with colorectal neoplasia could be given priority outpatient assessment and investigation and that 98% of those with a malignancy would be identified by the F.O.B. test. St John *et al.* (1990) have described a similar sensitivity (95%) for symptomatic cancers, although in their series 89% of patients also had a positive Haemoccult test.

It seems probable that the main reason for the enhanced sensitivity of Hemeselect for neoplasia is its ability to detect small quantities of faecal blood (Yoshida *et al.*, 1986), however it appears that lack of haemoglobin degradation may also adversely affect the performance of Haemoccult as the greatest difference in sensitivity between the tests was for rectal cancers.

The potential use of Hemeselect as a highly sensitive screening test for asymptomatic neoplasia is also raised by this study. Our results suggest that it would also be more sensitive than Haemoccult for asymptomatic neoplasia, but that this improvement would be achieved at the expense of specificity. The specificity of 84.1% for colorectal neoplasia achieved in the present study would be inadequate for a screening test although an improvement in specificity would be expected if the tests were used in asymptomatic individuals where blood loss from benign conditions would have less influence. In accordance with the United Kingdom Coordinating Committee for Cancer Research (UKCCCR) recommendations regarding the evaluation of F.O.B. tests a preliminary study to assess the overall positivity rate in asymptomatic subjects is now being undertaken and will be followed by a larger screening study to determine the comparative yield of neoplasia and specificity of Haemoccult and Hemeselect in asymptomatic subjects.

### References

ALDERCREUTZ, H., PARTANEN, P., VIRKOLA, P., LIEWENDAHL, K. & TURUNEN, M.J. (1984). Five guaiac – based tests for occult blood in faeces compared *in vitro* and *in vivo*. *Scand. J. Clin. Lab. Invest.*, **44**, 519.

BARRISON, I.G. & PARKINS, R.A. (1985). The clinical value of Haemoccult and fecatwin in the detection of colorectal neoplasia in hospital and general practice patients. *Postgrad. Med. J.*, **61**, 701.

FARRANDS, P.A. & HARDCASTLE, J.D. (1983). Accuracy of occult blood tests over a six-day period. *Clin. Oncol.*, **9**, 217.

HARDCASTLE, J.D., THOMAS, W.M., CHAMBERLAIN, J. & 7 others (1989). Randomized, controlled trial of faecal occult blood screening for colorectal cancer: results for first 107349 subjects. *Lancet*, **i**, 1160.

KEWENTER, J., BJORK, S., HAGLIND, E., SMITH, L., SVANVIK, J. & AHREN, C. (1988). Screening and rescreening for colorectal cancer; a controlled trial of faecal occult blood testing in 27,700 subjects. *Cancer*, **62**, 645.

KRONBORG, O., FENGER, C., OLSEN, J., BECH, K. & SONDERGAARD, O. (1989). Repeated screening for colorectal cancer with fecal occult blood test. A prospective randomized study at Funen, Denmark. *Scand. J. Gastroenterol.*, **24**, 599.

LEICESTER, R.J., LIGHTFOOT, A., MILLAR, J., COLIN-JONES, D.G. & HUNT, R.H. (1983). Accuracy and value of the Haemoccult test in symptomatic patients. *Br. Med. J.*, **286**, 673.

MANDEL, J.S., BOND, J.H., BRADLEY, M. & 7 others (1989). Sensitivity, specificity and positive predictivity of the Haemoccult test in screening for colorectal cancers. *Gastroenterology*, **97**, 597.

ST JOHN, D.J.B., YOUNG, G.P., CUTHBERTSON, A.M. & 5 others (1990). Detection of colorectal neoplasia: comparison of guaiac porphyrin and immunological tests for occult blood. *Gastroenterology*, **96**, A492.

THOMAS, W.M., PYE, G., HARDCASTLE, J.D., CHAMBERLAIN, J. & CHARNLEY, R.M. (1989). Haemoccult screening for colorectal cancer - The role of dietary restriction and selective three month rescreening. *Br. J. Surg.*, **76**, 976.

YOSHIDA, Y., SAITO, H., TSUCHIDA, S., KAKIZAKI, R., AISAWA, T. & MUNAKATA, A. (1986). A simple and sensitive immunological fecal occult blood test suitable for mass screening for colorectal cancer. *Gastroenterology*, **90**, 1699.